# Transcriptional Circuits Regulating Developmental Processes in *Candida albicans*

**DOI:** 10.3389/fcimb.2020.605711

**Published:** 2020-12-03

**Authors:** Diana L. Rodriguez, Morgan M. Quail, Aaron D. Hernday, Clarissa J. Nobile

**Affiliations:** ^1^ Department of Molecular and Cell Biology, School of Natural Sciences, University of California—Merced, Merced, CA, United States; ^2^ Quantitative and Systems Biology Graduate Program, University of California—Merced, Merced, CA, United States; ^3^ Health Sciences Research Institute, University of California - Merced, Merced, CA, United States

**Keywords:** *Candida albicans*, biofilms, commensal-pathogen transition, transcriptional regulation, transcriptional networks, transcriptional rewiring, white-opaque switching, transcriptional circuits

## Abstract

*Candida albicans* is a commensal member of the human microbiota that colonizes multiple niches in the body including the skin, oral cavity, and gastrointestinal and genitourinary tracts of healthy individuals. It is also the most common human fungal pathogen isolated from patients in clinical settings. *C. albicans* can cause a number of superficial and invasive infections, especially in immunocompromised individuals. The ability of *C. albicans* to succeed as both a commensal and a pathogen, and to thrive in a wide range of environmental niches within the host, requires sophisticated transcriptional regulatory programs that can integrate and respond to host specific environmental signals. Identifying and characterizing the transcriptional regulatory networks that control important developmental processes in *C. albicans* will shed new light on the strategies used by *C. albicans* to colonize and infect its host. Here, we discuss the transcriptional regulatory circuits controlling three major developmental processes in *C. albicans*: biofilm formation, the white-opaque phenotypic switch, and the commensal-pathogen transition. Each of these three circuits are tightly knit and, through our analyses, we show that they are integrated together by extensive regulatory crosstalk between the core regulators that comprise each circuit.

## Introduction

*C. albicans* is a common human commensal that asymptomatically colonizes the skin, oral cavity, and gastrointestinal and genitourinary tracts of healthy individuals (Kennedy and Volz, 1985; Kumamoto, 2002; Achkar and Fries, 2010; Spiliopoulou et al., 2010; Kumamoto, 2011; Nobile and Johnson, 2015; Kan et al., 2020). It is also an opportunistic pathogen that is capable of causing superficial mucosal and life-threatening disseminated infections, especially in immunocompromised individuals (Wenzel, 1995; Calderone and Fonzi, 2001; Hube, 2004; Pappas et al., 2004; Mayer et al., 2013), such as in AIDS, chemotherapy and organ transplant patients, as well as in individuals with implanted medical devices (Wenzel, 1995; Nobile and Johnson, 2015). Multiple regulatory pathways controlling important *C. albicans* developmental processes allow this opportunistic fungal pathogen to adapt to and proliferate in distinct environmental niches in the host. In this review, we discuss the “core” transcriptional circuits controlling three major developmental processes in *C. albicans*: biofilm formation, the white-opaque phenotypic switch, and the commensal-pathogen transition. The core circuitry is defined as the direct physical interactions between transcriptional regulators that control these developmental processes and their respective upstream intergenic regions, where at least one direct binding interaction with other members of the circuit has been experimentally observed. These three circuits were chosen because they regulate persistent phenotypic changes in *C. albicans* that have been characterized using genome-wide transcriptional profiling (RNA-sequencing and/or microarray) and binding (chromatin immunoprecipitation) approaches. In our discussion of these circuits we focus largely on transcription factors (TFs) that bind to DNA in a sequence-specific manner; however, we also include some discussion of important cofactors for which genome-wide transcriptional profiling and binding data are available. In addition, we include information on “auxiliary” transcriptional regulators of these three developmental processes that we define as those that are known to regulate these processes, but that lack direct binding interactions with the core transcriptional regulators or binding data is not available for these transcriptional regulators under the growth condition of interest.

## Regulation of Biofilm Formation

**Figure 1:**
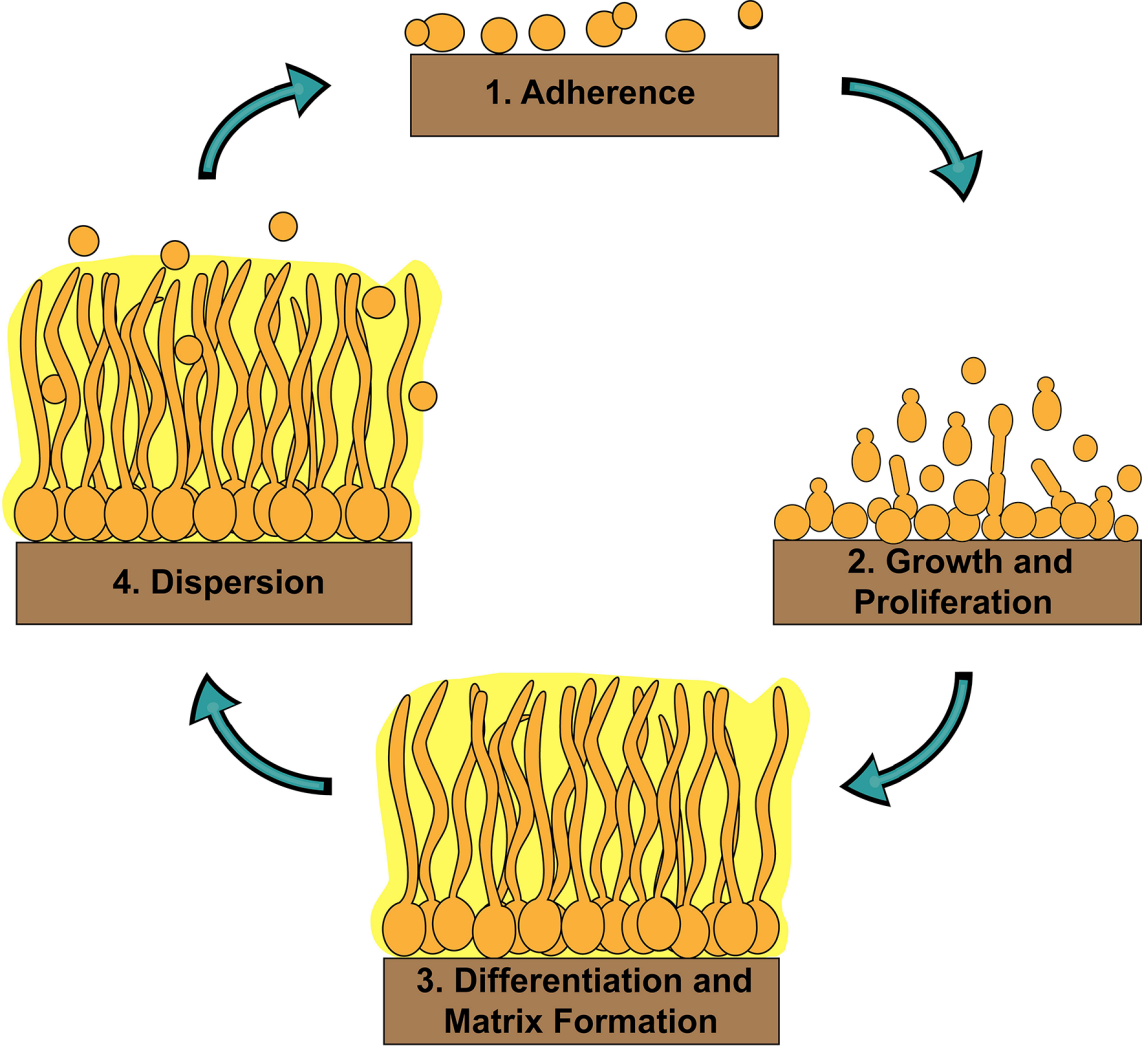
Stages of *C. albicans* biofilm formation. *C. albicans* biofilm formation occurs in four basic temporal stages: 1) adherence of yeast-form cells to a surface; 2) growth and proliferation of yeast-form cells forming a basal layer of anchoring cells; 3) differentiation of a proportion of yeast-form cells into hyphal cells and production of the extracellular matrix; and 4) dispersion of yeast-form cells out of the biofilm to cause bloodstream infections or to colonize new sites for biofilm formation.

Biofilms are communities of adherent microbial cells encased in protective extracellular matrices (Kolter and Greenberg, 2006; Nobile and Johnson, 2015; Gulati and Nobile, 2016). Biofilms are ubiquitous in nature and are typically associated with interfaces, such as solid-liquid, liquid-gas, and liquid-liquid interfaces (Davey and O’toole, 2000; Kolter and Greenberg, 2006; Wilking et al., 2011; Desai and Ardekani, 2020). They are problematic when they form in industrial settings, such as in water distribution systems and on food preparation settings, and even more so when they form inside a host on tissues and on implanted medical devices. *C. albicans* biofilms are composed of several cell types, including round budding yeast-form cells, oval pseudohyphal cells, and elongated hyphal cells, encased in a protective extracellular matrix (Chandra et al., 2001; Desai and Mitchell, 2015). *C. albicans* biofilm formation occurs in four basic temporal stages: i) adherence of yeast-form cells to a surface; ii) growth and proliferation of yeast-form cells forming a basal layer of anchoring cells; iii) differentiation of a proportion of yeast-form cells into hyphal cells and production of the extracellular matrix; and iv) dispersion of yeast-form cells out of the biofilm to cause bloodstream infections or to colonize new sites for biofilm formation (**Figure 1**) (Desai and Mitchell, 2015; Nobile and Johnson, 2015; Gulati and Nobile, 2016). Indeed, *C. albicans* is a common cause of bloodstream infections worldwide, which often originate from biofilms (Edmond et al., 1999; Richards et al., 1999; Pfaller and Diekema, 2007). Given that cells within *C. albicans* biofilms are inherently resistant and tolerant to most antifungal drug treatments compared to planktonic (free-floating) cells, biofilm infections are particularly challenging to treat in the clinic. Understanding the genetic regulatory mechanisms that control *C. albicans* biofilm formation could lead to the development of novel therapeutic strategies effective in treating biofilm infections.

The *C. albicans* transcriptional network controlling biofilm formation was first described eight years ago (Nobile et al., 2012). Six “master” biofilm transcriptional regulators (Bcr1, Tec1, Efg1, Ndt80, Rob1, and Brg1) were identified by screening a library of 165 transcription factor (TF) mutant strains (Homann et al., 2009) for defects in biofilm formation under standard *in vitro* biofilm growth conditions (Nobile et al., 2012). Here, we define a master biofilm transcriptional regulator as one whose deletion impairs biofilm formation throughout a 48-h period of biofilm growth under these standard conditions. All six TF mutant strains identified additionally had clear defects in biofilm formation in at least one of two *in vivo* animal models for biofilm formation (Nobile et al., 2012). Using genome-wide transcriptional profiling and chromatin immunoprecipitation techniques to study mature 48-h biofilms, a complex interconnected transcriptional network was discovered consisting of those six master transcriptional regulators, along with 1,061 downstream “target” genes (Nobile et al., 2012). These six master transcriptional regulators directly bound to the upstream intergenic regions and positively regulated the expression of each other, forming a tightly knit core biofilm circuit (Fox and Nobile, 2012; Nobile et al., 2012). Additionally, with the exception of Tec1, all of the six master biofilm transcriptional regulators acted as both repressors and activators of their directly bound biofilm target genes; Tec1, on the other hand, primarily acted as an activator (Nobile et al., 2012). Each of the six master biofilm transcriptional regulators controlled target genes that were in common with the other core transcriptional regulators in the circuit, as well as target genes that were unique to each transcriptional regulator. These findings suggest that each master biofilm transcriptional regulator in the circuit controls certain elements of biofilm formation independently, but that they also work together to coordinate concerted efforts important for biofilm formation. For example, Ndt80 regulates the expression of drug transporters independent of the other master biofilm transcriptional regulators in the circuit (such as, *CDR4*), and some in common with several of the other master biofilm transcriptional regulators in the circuit (such as, *CDR3*) (Nobile et al., 2012). Additionally, each master biofilm transcriptional regulator likely responds to unique environmental inputs, such as oxygen and nutrient availability, pH, temperature, and waste products. How different environmental inputs influence the biofilm transcriptional circuit is an intriguing area of future research. For example, we know that the six master biofilm transcriptional regulators discovered using *in vitro* biofilm assays are still required for *in vivo* biofilm formation in at least one of two *in vivo* biofilm models (Nobile et al., 2012). The majority (four) of the master biofilm transcriptional regulators discovered in this study were essential for biofilm formation in both *in vivo* biofilm models used; however, two of the master biofilm transcriptional regulators played different roles depending on the *in vivo* biofilm model (Nobile et al., 2012). Specifically, Bcr1 was essential for biofilm formation in a rat catheter biofilm model but was dispensable in a rat denture biofilm model (Nobile et al., 2012). Similarly, Brg1 was essential for biofilm formation in a rat denture biofilm model but was dispensable in a rat catheter biofilm model (Nobile et al., 2012). Future work on these master transcriptional regulators will determine their unique influences on biofilm formation dependent on the environmental inputs present.

**Figure 2:**
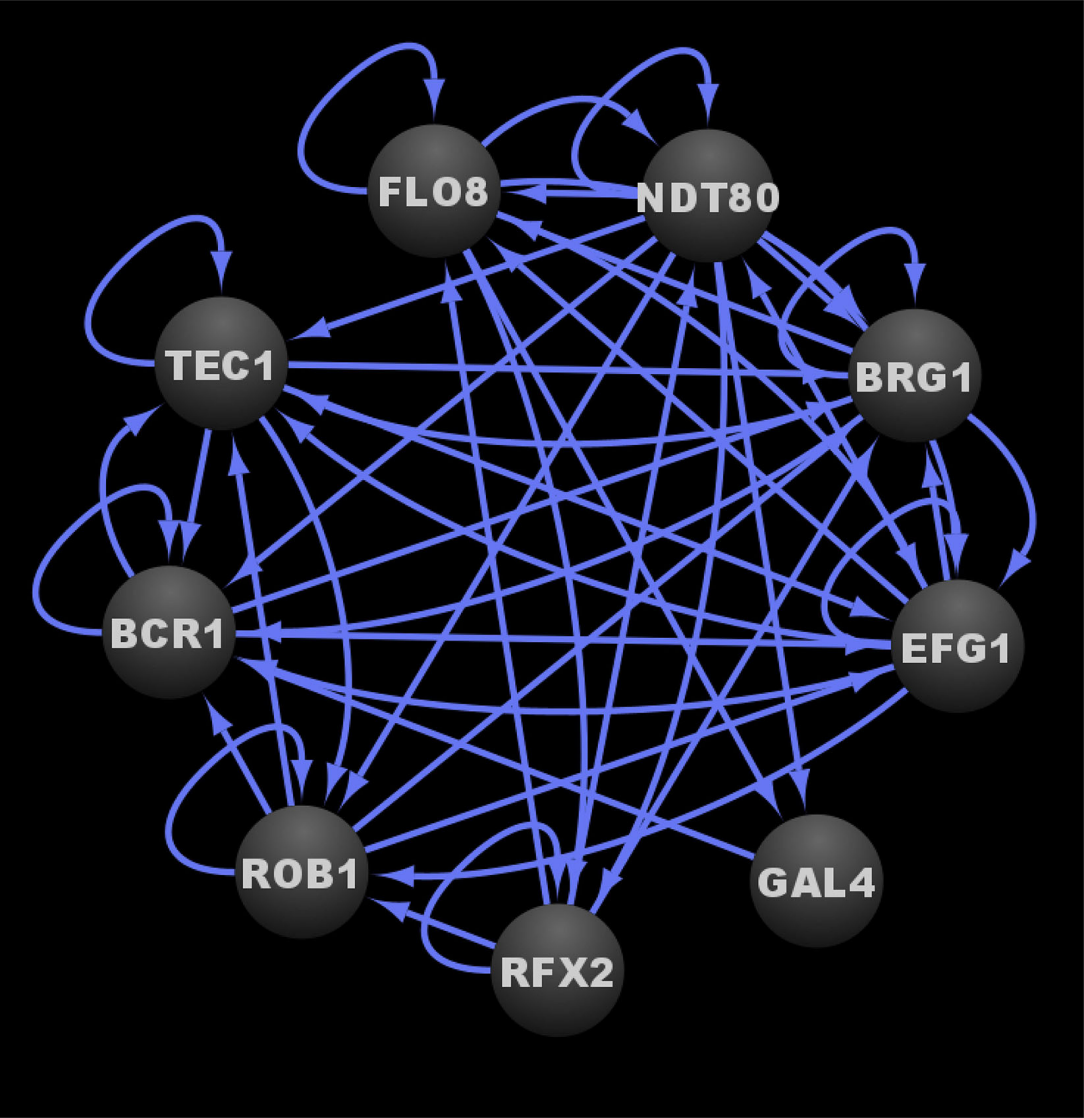
Transcriptional circuit controlling *C. albicans* biofilm formation. Ovals indicate each of the core biofilm transcriptional regulators with their respective names. Arrows indicate direct binding events. See **Data Sheet S1, Tab4** for binding interactions. Data were derived from (Nobile et al., 2012; Fox et al., 2015). Figure was generated using Cytoscape (Shannon et al., 2003).

In a subsequent study, three additional transcriptional regulators, Gal4, Rfx2, and Flo8, were added to the core biofilm transcriptional circuit (Fox et al., 2015). Gal4, Rfx2, and Flo8 were found to directly bind to the upstream intergenic regions of one or more of the previously identified six master biofilm transcriptional regulators and vice versa during biofilm development (Nobile et al., 2012; Fox et al., 2015). Gal4, Rfx2, and Flo8 were identified (in addition to the six previously identified transcriptional regulators) by screening a TF mutant library containing 192 TF mutant strains (Fox et al., 2015). This TF library contained the same 165 TF mutants (Homann et al., 2009) from the Nobile et al., 2012 study (Nobile et al., 2012) plus 27 additional newly constructed TF mutant strains. The TF mutants in this larger library were screened for their abilities to form biofilms over time at 90 min, 8, 24, and 48 h of biofilm growth (Fox et al., 2015). Flo8, like the other six previously identified master biofilm transcriptional regulators, was required for biofilm formation throughout a 48-h course of biofilm growth, and thus was deemed to be a master biofilm transcriptional regulator; Gal4 and Rfx2 were only required for normal biofilm formation at specific intermediate time points (Fox et al., 2015). Given that the initial biofilm circuit consisting of six master transcriptional regulators was discovered by assessing biofilm formation at a single mature time point (48 h) (Nobile et al., 2012), performing the genetic screen as a biofilm develops over time, with the additional TF mutant strains, contributed to the expansion of the core biofilm circuit (Fox et al., 2015). Genome-wide binding data was not performed for Gal4, Rfx2, and Flo8 as part of this study; however, directed chromatin immunoprecipitation followed by quantitative PCR was performed to determine that these three new transcriptional regulators are integrated into the core biofilm circuit, which now consists of nine core transcriptional regulators, seven of which are considered to be master biofilm transcriptional regulators (**Figure 2**) (Nobile et al., 2012; Fox et al., 2015). We note that although genome-wide binding experiments have been performed for Gal4 and Flo8 (Askew et al., 2009; Polvi et al., 2019), these experiments were not performed under biofilm conditions and thus the resulting data cannot be integrated into the biofilm transcriptional circuit. Overall, although the logic of the biofilm transcriptional circuit (defined as how each transcriptional regulator contributes to the regulatory dynamics of the circuit) has yet to be fully elucidated, the high degree of interconnectivity between the core biofilm transcriptional regulators likely contributes to the robustness, yet reversibility, of the biofilm state.

Although the nine core biofilm transcriptional regulators are known to be important for biofilm formation, how each one specifically contributes to biofilm processes (e.g. adhesion, filamentation, antifungal drug resistance, etc.), through detailed analyses of their mutant strains, has not been systematically determined. **Table 1** summarizes the current knowledge of the roles of all known transcriptional regulators in known biofilm-related processes. Eight of the nine core biofilm transcriptional regulators (Bcr1, Brg1, Efg1, Flo8, Ndt80, Rfx2, Rob1, and Tec1) have been implicated in regulating filamentation (Schweizer et al., 2000; Bockmüh and Ernst, 2001; Cao et al., 2006; Elson et al., 2009; Hao et al., 2009; Sellam et al., 2010; Vandeputte et al., 2011; Du et al., 2012b; Nobile et al., 2012), which is a critical process necessary for maintaining the architectural stability of the biofilm structure. Four of the nine core biofilm transcriptional regulators (Bcr1, Efg1, Rfx2, and Tec1) have been implicated in regulating adhesion (Dieterich et al., 2002; Hao et al., 2009; Sahni et al., 2010; Finkel et al., 2012), including both cell-cell and cell-substrate adhesion, which is an essential process for both the initiation of biofilm formation as well as for the maintenance of a mature biofilm. Three of the nine core biofilm transcriptional regulators (Bcr1, Efg1, and Ndt80) are known to be involved in the regulation of antifungal drug resistance and/or tolerance (Chen et al., 2004; Sellam et al., 2009; Prasad et al., 2010; Desai et al., 2013), an important feature that contributes to the overall recalcitrance of established biofilms to antimicrobial compounds. Of the nine core biofilm transcriptional regulators, we know the least about the biofilm specific roles of Gal4, and only that it contributes to the structure of a biofilm at intermediate stages of biofilm development (Fox et al., 2015). In the future, additional roles of the nine core biofilm transcriptional regulators during biofilm formation will certainly be elucidated. For example, it seems likely that some of the core biofilm transcriptional regulators would be involved in the formation of the extracellular matrix; however, this role has not been examined to date in the mutant strains of the core biofilm transcriptional regulators. In addition, the ability of cells within biofilms to communicate with one another, called quorum sensing, is an important process for coordinating biofilm formation of many microorganisms; however, this role has yet to be examined in the mutant strains of the core biofilm transcriptional regulators. In fact, little is known in general on the regulation of quorum sensing during *C. albicans* biofilm development.

**Table 1:** Known transcriptional regulators with roles in *C. albicans* biofilm formation.

Core Biofilm Transcriptional Regulators				
Orf19#	Name	Known biofilm-related process affected in mutant strain	Gene upstream intergenic region bound by one or more of the core biofilm regulators?	References
Orf19.723	Bcr1	Adhesion, Filamentation, Drug Resistance/Tolerance	Yes	(Nobile and Mitchell, 2005; Elson et al., 2009; Homann et al., 2009; Fanning et al., 2012; Finkel et al., 2012; Desai et al., 2013)
Orf19.4056	Brg1	Filamentation	Yes	(Du et al., 2012b; Nobile et al., 2012)
Orf19.610	Efg1	Adhesion, Filamentation, Drug Resistance/Tolerance	Yes	(Bockmüh and Ernst, 2001; Dieterich et al., 2002; Ramage et al., 2002; Li and Palecek, 2003; Prasad et al., 2010; Nobile et al., 2012)
Orf19.1093	Flo8	Filamentation	Yes	(Cao et al., 2006; Fox et al., 2015)
Orf19.5338	Gal4	Unknown	Yes	(Fox et al., 2015)
Orf19.2119	Ndt80	Filamentation, Drug Resistance	Yes	(Chen et al., 2004; Sellam et al., 2009; Sellam et al., 2010; Nobile et al., 2012)
Orf19.4590	Rfx2	Adhesion, Filamentation	Yes	(Hao et al., 2009; Fox et al., 2015)
Orf19.4998	Rob1	Filamentation	Yes	(Vandeputte et al., 2011)
Orf19.5908	Tec1	Adhesion, Filamentation	Yes	(Schweizer et al., 2000; Staib et al., 2004; Nobile and Mitchell, 2005; Sahni et al., 2010)
**Auxiliary Biofilm Transcriptional Regulators**				
Orf19.6124	Ace2	Adhesion, Filamentation, Drug Resistance/Tolerance	No	(Kelly et al., 2004; Mulhern et al., 2006; Finkel et al., 2012)
Orf19.2331	Ada2	Adhesion, Filamentation, Drug Resistance/Tolerance	No	(Bruno et al., 2006; Pukkila-Worley et al., 2009; Finkel et al., 2012)
Orf19.7381	Ahr1	Adhesion, Filamentation, Drug Resistance/Tolerance	Yes	(Homann et al., 2009; Askew et al., 2011)
Orf19.4766	Arg81	Adhesion, Filamentation, Drug Resistance/Tolerance	No	(Homann et al., 2009; Finkel et al., 2012)
Orf19.6874	Bpr1	Unknown	Yes	(Fox et al., 2015)
Orf19.4670	Cas5	Adhesion, Drug Resistance/Tolerance	Yes	(Finkel et al., 2012; Vasicek et al., 2014)
Orf19.2356	Crz2	Adhesion, Drug Resistance/Tolerance	Yes	(Homann et al., 2009; Finkel et al., 2012)
Orf19.3127	Czf1	Adhesion, Filamentation, Drug Resistance/Tolerance	Yes	(Brown et al., 1999; Finkel et al., 2012; Langford et al., 2013)
Orf19.3252	Dal81	Adhesion	No	(Finkel et al., 2012)
Orf19.3193	Fcr3	Adhesion	Yes	(Finkel et al., 2012)
Orf19.6680	Fgr27	Adhesion, Filamentation	No	(Uhl et al., 2003; Finkel et al., 2012)
Orf19.1358	Gcn4	Filamentation	Yes	(García-Sánchez et al., 2004; Kamthan et al., 2012)
Orf19.4000	Grf10	Adhesion, Filamentation	Yes	(Ghosh et al., 2015)
Orf19.2842	Gzf3	Adhesion, Drug Resistance/Tolerance	Yes	(Homann et al., 2009; Fox et al., 2015)
Orf19.4225	Leu3	Adhesion	No	(Finkel et al., 2012)
Orf19.5312	Met4	Adhesion	No	(Finkel et al., 2012)
Orf19.4318	Mig1	Filamentation, Drug Resistance/Tolerance	Yes	(Homann et al., 2009; Lagree et al., 2020)
Orf19.5326	Mig2	Filamentation	No	(Lagree et al., 2020)
Orf19.6309	Mss11	Adhesion, Filamentation	Yes	(Tsai et al., 2014)
Orf19.2012	Not3	Adhesion, Filamentation	No	(Cheng et al., 2003; Finkel et al., 2012)
Orf19.7150	Nrg1	Filamentation, Drug Resistance/Tolerance, Dispersion	Yes	(Wheeler et al., 2008; Uppuluri et al., 2010b)
Orf19.4093	Pes1	Filamentation, Drug Resistance/Tolerance, Dispersion	No	(Xu et al., 2007; Shen et al., 2008; Uppuluri et al., 2010a)
Orf19.2823	Rfg1	Adhesion, Filamentation	Yes	(Kadosh and Johnson, 2001; Fox et al., 2015)
Orf19.1604	Rha1	Filamentation	Yes	(Omran et al., 2020)
Orf19.7247	Rim101	Adhesion, Filamentation, Drug Resistance/Tolerance	Yes	(Cornet et al., 2006; Fox et al., 2015)
Orf19.4662	Rlm1	Drug Resistance/Tolerance, Extracellular Matrix Production	No	(Nett et al., 2011; Delgado-Silva et al., 2014)
Orf19.5953	Sfp1	Adhesion	Yes	(Chen and Lan, 2015)
Orf19.5871	Snf5	Adhesion	No	(Finkel et al., 2012)
Orf19.4961	Stp2	Adhesion, Filamentation	Yes	(Böttcher et al., 2020)
Orf19.7319	Suc1	Adhesion	No	(Finkel et al., 2012)
Orf19.798	Taf14	Adhesion, Filamentation	No	(Finkel et al., 2012; Wang et al., 2020)
Orf19.4062	Try2	Adhesion	No	(Finkel et al., 2012)
Orf19.1971	Try3	Adhesion	No	(Finkel et al., 2012)
Orf19.5975	Try4	Adhesion	Yes	(Finkel et al., 2012)
Orf19.3434	Try5	Adhesion	Yes	(Finkel et al., 2012)
Orf19.6824	Try6	Adhesion	Yes	(Finkel et al., 2012)
Orf19.4941	Tye7	Filamentation	Yes	(Bonhomme et al., 2011)
Orf19.7317	Uga33	Adhesion	No	(Finkel et al., 2012)
Orf19.1822	Ume6	Filamentation, Dispersion	Yes	(Uppuluri et al., 2010a; Uppuluri et al., 2010b)
Orf19.391	Upc2	Adhesion, Drug Resistance/Tolerance	No	(Silver et al., 2004; Dunkel et al., 2008; Kakade et al., 2019)
Orf19.1035	War1	Adhesion	No	(Finkel et al., 2012)
Orf19.3794	Zap1	Filamentation, Extracellular Matrix Production	Yes	(Kim et al., 2008b; Nobile et al., 2009; Ganguly et al., 2011; Finkel et al., 2012)
Orf19.1718	Zcf8	Adhesion	Yes	(Finkel et al., 2012)
Orf19.4767	Zcf28	Adhesion	No	(Finkel et al., 2012)
Orf19.5924	Zcf31	Adhesion	Yes	(Finkel et al., 2012)
Orf19.5940	Zcf32	Adhesion, Filamentation	No	(Kakade et al., 2016; Kakade et al., 2019)
Orf19.6182	Zcf34	Adhesion, Drug Resistance/Tolerance	No	(Homann et al., 2009; Oh et al., 2010; Finkel et al., 2012)
Orf19.7583	Zcf39	Adhesion	No	(Finkel et al., 2012)
Orf19.6781	Zfu2	Adhesion, Drug Resistance/Tolerance	No	(Finkel et al., 2012; Vandeputte et al., 2012)
Orf19.3187	Znc1	Adhesion	No	(Finkel et al., 2012)

In addition to these nine transcriptional regulators that make up the core biofilm circuit, there are 50 “auxiliary” transcriptional regulators that have been implicated in biofilm formation (**Table 1**). The majority of these auxiliary biofilm transcriptional regulators are also bound in their upstream intergenic regions by at least one of the initial six master biofilm transcriptional regulators (Bcr1, Tec1, Efg1, Ndt80, Rob1, or Brg1; note that of the nine core biofilm transcriptional regulators, there is not genome-wide chromatin immunoprecipitation data available for Gal4, Rfx2, and Flo8, and thus we do not know whether they bind to the auxiliary biofilm transcriptional regulators) (**Table 1**) (Nobile et al., 2012). As such, several of the 50 auxiliary transcriptional regulators are integrated into the larger biofilm network that includes the core nine transcriptional regulators and all of their directly bound target genes (Nobile et al., 2012). Based on existing phenotypic analyses of the mutant strains of the auxiliary biofilm transcriptional regulators, the majority (48) are implicated in the regulation of adhesion and/or filamentation (Brown et al., 1999; Kadosh and Johnson, 2001; Cheng et al., 2003; Uhl et al., 2003; García-Sánchez et al., 2004; Kelly et al., 2004; Mulhern et al., 2006; Kim et al., 2008b; Shen et al., 2008; Wheeler et al., 2008; Homann et al., 2009; Nobile et al., 2009; Pukkila-Worley et al., 2009; Uppuluri et al., 2010a; Uppuluri et al., 2010b; Askew et al., 2011; Bonhomme et al., 2011; Ganguly et al., 2011; Finkel et al., 2012; Kamthan et al., 2012; Langford et al., 2013; Delgado-Silva et al., 2014; Tsai et al., 2014; Chen and Lan, 2015; Fox et al., 2015; Ghosh et al., 2015; Kakade et al., 2016; Kakade et al., 2019; Böttcher et al., 2020; Lagree et al., 2020; Omran et al., 2020; Wang et al., 2020); 16 are implicated in drug resistance and/or tolerance (Bruno et al., 2006; Cornet et al., 2006; Mulhern et al., 2006; Xu et al., 2007; Dunkel et al., 2008; Wheeler et al., 2008; Homann et al., 2009; Prasad et al., 2010; Nett et al., 2011; Vandeputte et al., 2012; Langford et al., 2013; Vasicek et al., 2014); two are implicated in the production of the extracellular matrix (Finkel et al., 2012; Delgado-Silva et al., 2014); and two are implicated in dispersion (Uppuluri et al., 2010b; Uppuluri et al., 2010a). Similar to the core biofilm transcriptional regulators, detailed analyses of the mutant strains of the auxiliary biofilm transcriptional regulators have not been systemically studied for known biofilm processes. Rather, most of their roles in biofilm processes have been determined through large-scale genetic screens. Of the auxiliary biofilm transcriptional regulators, we understand the least about the biofilm specific roles of Bpr1/Orf19.6874, which is only known to contribute to biofilm biomass throughout biofilm development (Fox et al., 2015). Future detailed phenotypic analyses of the auxiliary transcriptional regulator mutant strains in biofilm specific processes will certainly reveal new and additional roles for these transcriptional regulators in biofilm development.

## Regulation of the White-Opaque Phenotypic Switch

The white-opaque switch in *C. albicans* is a form of phenotypic switching that gives rise to two distinct cell types called “white” and “opaque” that display distinct phenotypic characteristics at the single cell and colony levels (Anderson and Soll, 1987; Slutsky et al., 1987; Rikkerink et al., 1988; Bergen et al., 1990; Soll, 1992; Soll et al., 1993). White cells represent the standard budding yeast form of *C. albicans*, forming shiny, white, dome-shaped colonies on solid media plates, while opaque cells are larger and more elongated than white cells and form dull, off-white, flattened colonies on solid media plates (Slutsky et al., 1987; Soll et al., 1993; Lohse and Johnson, 2009; Noble et al., 2017). White and opaque cells differ in their virulence characteristics, metabolic preferences, mating competencies, interactions with the host innate immune system, and responses to environmental stimuli (Kolotila and Diamond, 1990; Lan et al., 2002; Lockhart et al., 2002; Miller and Johnson, 2002; Bennett et al., 2003; Geiger et al., 2004; Dumitru et al., 2007; Lohse and Johnson, 2008; Ramírez-Zavala et al., 2008; Huang et al., 2009; Huang et al., 2010; Lohse et al., 2013; Xie et al., 2013; Lohse et al., 2016; Du and Huang, 2016; Ene et al., 2016; Dalal et al., 2019). In total, nearly 20% of the transcriptome is differentially expressed, by at least twofold, between the two cell types, highlighting that the white-opaque switch involves major transcriptional rewiring (Tuch et al., 2010; Hernday et al., 2013). Under standard switch permissive growth conditions, switching between the white cell type, considered the “ground” state, and the opaque cell type, considered the “excited” state, occurs stochastically at a frequency of roughly one switch event per 1,000-10,000 cell divisions (Rikkerink et al., 1988; Bergen et al., 1990; Ramírez-Zavala et al., 2008; Alby and Bennett, 2009b). Each cell type is heritably maintained without any change to the primary sequence of the genome, thus fitting the classic definition of an epigenetic switch (Slutsky et al., 1987; Soll et al., 1993; Zordan et al., 2006; Zordan et al., 2007). The switch is responsive to the combined effects of environmental signals, such as carbon source, pH, CO_2_ levels, and temperature, which can differentially bias the cell population towards one of the two cell types (Dumitru et al., 2007; Ramírez-Zavala et al., 2008; Alby and Bennett, 2009a; Huang et al., 2009; Huang, 2012; Lohse et al., 2013; Du and Huang, 2016; Ene et al., 2016; Dalal et al., 2019). Mating type can also influence the ability of the cells to undergo white-opaque switching, where *MTL* heterozygous (**a**/α) cells are typically “locked” in the white state, while *MTL* hemizygous (**a**/Δ, α/Δ), homozygous (**a**/**a**, or α/**α**), and haploid (**a** or α) cells are capable of undergoing stochastic white-opaque switching (Hull and Johnson, 1999; Lockhart et al., 2002; Miller and Johnson, 2002). This mating type dependency, however, is not exclusive to all strains; in fact, a significant fraction of *MTL* heterozygous clinical isolates can be induced to form opaque cells under specific growth conditions that promote white to opaque switching in *MTL* hemizygous, homozygous, or haploid cells (Xie et al., 2013).

**Figure 3:**
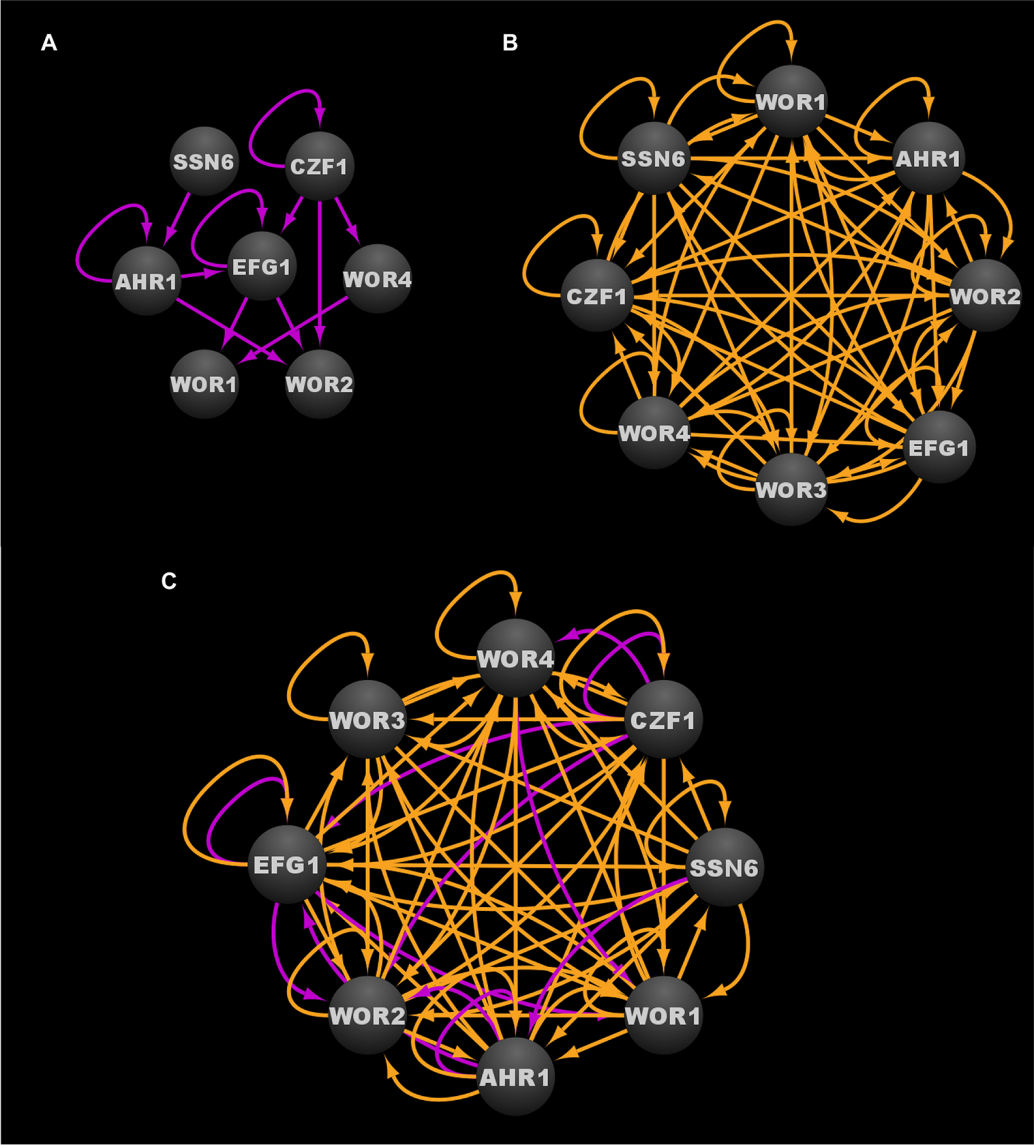
Transcriptional circuits controlling the *C. albicans* white-opaque phenotypic switch. **(A)** Transcriptional circuit of the white state. **(B)** Transcriptional circuit of the opaque state. **(C)** Overlayed transcriptional circuits regulating the white and opaque states. Ovals indicate each of the core regulators with their respective names. Arrows indicate direct binding events. See **Data Sheet S1**, **Tab4** for binding interactions. Data were derived from (Zordan et al., 2007; Hernday et al., 2013; Lohse et al., 2013; Hernday et al., 2016; Lohse and Johnson, 2016). Figure was generated using Cytoscape (Shannon et al., 2003).

Through a combination of forward and reverse genetic approaches, a total of 112 transcriptional regulators and one protein binding cofactor (Ssn6) have been identified which, when deleted, significantly impact the frequency of white-opaque switching (**Table 2**) (Huang et al., 2006; Srikantha et al., 2006; Zordan et al., 2006; Zordan et al., 2007; Hernday et al., 2013; Lohse et al., 2013; Du et al., 2015; Hernday et al., 2016; Lohse et al., 2016; Lohse and Johnson, 2016). Of these 113 switch regulating proteins, eight (Wor1, Wor2, Wor3, Wor4, Czf1, Efg1, Ahr1, and Ssn6) are considered to be core switch regulators, and have been extensively characterized by genome-wide transcriptional profiling and chromatin immunoprecipitation approaches in white and opaque cell types; the remaining 105 switch regulating proteins are considered to be auxiliary switch regulators (**Table 2**) (Sonneborn et al., 1999; Srikantha et al., 2000; Huang et al., 2006; Zordan et al., 2006; Zordan et al., 2007; Vinces and Kumamoto, 2007; Lohse and Johnson, 2010; Wang et al., 2011; Hernday et al., 2013; Lohse et al., 2013; Lohse and Johnson, 2016; Hernday et al., 2016). Together, these eight core switch regulators form complex cell type specific networks, with 203 bound target genes in white cells and 756 bound target genes in opaque cells (Hernday et al., 2013; Lohse et al., 2013; Hernday et al., 2016; Lohse and Johnson, 2016). At the center of the white and opaque specific regulatory networks are two distinct transcriptional circuits (see **Figure 3A** for the white circuit, **Figure 3B** for the opaque circuit, and **Figure 3C** for the combined white and opaque overlayed circuits) that consist of interconnected positive and negative feedback loops that govern the cell fate and heritable maintenance of the white and opaque cell types (Vinces et al., 2006; Vinces and Kumamoto, 2007; Zordan et al., 2007; Hernday et al., 2013; Hernday et al., 2016; Lohse and Johnson, 2016). Although several groups have identified kinases, chromatin modifiers, and other proteins that also affect white-opaque switching (Hnisz et al., 2009; Noble et al., 2017; Rai et al., 2018); here, we focus on the eight core switch regulators (TFs: Wor1, Wor2, Wor3, Wor4, Czf1, Efg1, Ahr1; and cofactor: Ssn6) for which genome-wide transcriptional profiling and chromatin immunoprecipitation data are available.

**Table 2:** Known transcriptional regulators and a protein cofactor with roles in the *C. albicans* white-opaque switch^‡^.

Core White-Opaque Transcriptional Regulators and a Protein Cofactor				
Orf19#	Name	Known effect on white-opaque switch in mutant strain*		Gene upstream intergenic bound by one or more of the core white-opaque regulators?
		White to Opaque	Opaque to White		
Orf19.7381	Ahr1	2.0	-7.8	Yes
Orf19.3127	Czf1	-21.9	-16.8	Yes
Orf19.610	Efg1	24.0	-62.7	Yes
Orf19.6798	Ssn6	N/A	N/A	Yes
Orf19.4884	Wor1	-20.8	N/A	Yes
Orf19.5992	Wor2	-32.9	N/A	Yes
Orf19.467	Wor3	-2.4	-3.9	Yes
Orf19.6713	Wor4	-13.3	N/A	Yes
**Auxiliary White-Opaque Transcriptional Regulators**				
Orf19.7436	Aaf1	-1.1	-2.7	Yes
Orf19.2272	Aft2	-2.8	-1.7	Yes
Orf19.4766	Arg81	1.8	-2.3	Yes
Orf19.166	Asg1	-21.6	-22.1	Yes
Orf19.5343	Ash1	-1.2	-26.9	Yes
Orf19.6874	Bas1	-1.5	2.5	Yes
Orf19.723	Bcr1	2.2	N/A	Yes
Orf19.4056	Brg1	1.9	-1.5	Yes
Orf19.1623	Cap1	-1.5	-4.4	Yes
Orf19.4670	Cas5	1.4	-2.3	Yes
Orf19.4433	Cph1	-2.2	-2.6	Yes
Orf19.1187	Cph2	-2.3	-1.4	No
Orf19.7359	Crz1	1.9	-5.6	Yes
Orf19.3794	Csr1	1.0	2.6	Yes
Orf19.7374	Cta4	-1.1	-5.9	Yes
Orf19.4288	Cta7	2.4	-2.1	Yes
Orf19.5001	Cup2	-1.2	-1.6	Yes
Orf19.6514	Cup9	4.7	-15.4	Yes
Orf19.3252	Dal81	-6.1	-1.8	Yes
Orf19.2088	Dpb4	-3.1	-2.4	Yes
Orf19.2623	Ecm22	1.3	-2.2	Yes
Orf19.5498	Efh1	1.7	-1.6	Yes
Orf19.6817	Fcr1	-1.9	-1.6	Yes
Orf19.2054	Fgr15	-17.7	4.8	Yes
Orf19.1093	Flo8	-27.8	N/A	No
Orf19.5338	Gal4	-23.9	-1.3	Yes
Orf19.3182	Gis2	-1.2	-9.4	Yes
Orf19.4000	Grf10	1.4	-6.9	Yes
Orf19.2842	Gzf3	-12.3	1.7	Yes
Orf19.1228	Hap2	-28.6	-1.6	No
Orf19.4647	Hap3	-3.0	1.3	Yes
Orf19.517	Hap31	-22.7	-1.3	Yes
Orf19.740	Hap41	-9.6	1.1	Yes
Orf19.1481	Hap42	-2.0	-1.9	No
Orf19.1973	Hap5	-6.7	-3.0	Yes
Orf19.4853	Hcm1	18.3	-3.7	Yes
Orf19.3063	Hfl1	-21.0	2.1	Yes
Orf19.7539	Ino2	-23.5	-3.6	Yes
Orf19.837.1	Ino4	-3.0	-1.2	Yes
Orf19.7401	Isw2	3.4	2.9	Yes
Orf19.3736	Kar4	-2.0	1.2	Yes
Orf19.4776	Lys143	7.3	-1.1	Yes
Orf19.5380	Lys144	1.3	-2.4	Yes
Orf19.7068	Mac1	-19.4	-1.6	Yes
Orf19.4318	Mig1	-29.7	1.4	Yes
Orf19.5326	Mig2	1.6	-1.6	Yes
Orf19.4752	Msn4	1.9	-4.7	Yes
Orf19.2119	Ndt80	-10.1	1.9	Yes
Orf19.5910	Nto1	2.8	-1.5	Yes
Orf19.1543	Opi1	4.0	-2.4	Yes
Orf19.4231	Pth2	3.0	-4.1	Yes
Orf19.1773	Rap1	16.0	-1.6	Yes
Orf19.5558	Rbf1	N/A	-32.4	Yes
Orf19.6102	Rca1	-9.1	-1.9	Yes
Orf19.7521	Rep1	-1.5	2.4	Yes
Orf19.2823	Rfg1	1.1	2.1	Yes
Orf19.3865	Rfx1	1.8	1.7	Yes
Orf19.4590	Rfx2	1.5	-1.7	Yes
Orf19.1604	Rha1	1.0	-2.7	Yes
Orf19.4438	Rme1	2.1	-1.8	Yes
Orf19.513	Ron1	-1.2	-1.7	Yes
Orf19.1069	Rpn4	18.2	-1.5	No
Orf19.4722	Rtg1	-2.1	-2.9	Yes
Orf19.2315	Rtg3	-2.8	-2.2	Yes
Orf19.1926	Sef2	1.1	-3.3	Yes
Orf19.454	Sfl1	-1.1	2.0	Yes
Orf19.971	Skn7	1.1	-1.5	Yes
Orf19.1032	Sko1	-1.8	-2.4	No
Orf19.4961	Stp2	9.2	-6.9	Yes
Orf19.909	Stp4	3.3	-2.2	Yes
Orf19.4545	Swi4	-4.5	1.0	Yes
Orf19.4941	Tye7	2.0	-1.0	Yes
Orf19.7317	Uga33	-1.0	-1.7	Yes
Orf19.1822	Ume6	-1.6	2.0	Yes
Orf19.2745	Ume7	-2.0	1.4	Yes
Orf19.391	Upc2	-1.1	3.1	Yes
Orf19.1035	War1	-3.2	-1.1	No
Orf19.5210	Xbp1	-6.4	-1.2	Yes
Orf19.2808	Zcf16	1.5	1.2	Yes
Orf19.3305	Zcf17	1.3	2.2	Yes
Orf19.431	Zcf2	-1.7	-2.8	Yes
Orf19.4145	Zcf20	-1.5	-2.2	Yes
Orf19.4166	Zcf21	-4.1	-40.4	Yes
Orf19.4251	Zcf22	1.8	-1.1	Yes
Orf19.4524	Zcf24	-1.0	-3.2	Yes
Orf19.4568	Zcf25	8.5	-2.9	Yes
Orf19.4649	Zcf27	-1.9	1.5	Yes
Orf19.5251	Zcf30	1.1	-1.7	Yes
Orf19.5924	Zcf31	-2.4	3.2	Yes
Orf19.6182	Zcf34	-2.9	-4.6	Yes
Orf19.1685	Zcf7	4.7	-2.7	Yes
Orf19.1718	Zcf8	-2.1	-2.2	Yes
Orf19.6781	Zfu2	-1.9	2.3	Yes
Orf19.6888	Zfu3	-5.0	-16.2	Yes
Orf19.5026	Zms1	-2.8	-1.2	Yes
Orf19.1150		1.2	-1.3	No
Orf19.1274		-1.4	1.2	No
Orf19.1577		-1.1	-1.5	No
Orf19.1757		1.0	-1.6	Yes
Orf19.217		-1.7	-1.7	Yes
Orf19.2476		1.9	2.5	Yes
Orf19.2612		2.4	1.4	Yes
Orf19.2961		7.0	2.0	Yes
Orf19.3928		5.7	-4.4	Yes
Orf19.7098		7.8	1.1	Yes

**^‡^**Data derived from (Zordan et al., 2007; Hernday et al., 2013; Lohse et al., 2013; Hernday et al., 2016; Lohse et al., 2016; Lohse and Johnson, 2016). *Fold change in switch frequency is relative to a wildtype reference strain.

Wor1 is considered to be the master regulator of the white-opaque switch, as it is the only switch regulator that is known to be required for both the transition to, and heritable maintenance of, the opaque cell type (Huang et al., 2006; Srikantha et al., 2006; Zordan et al., 2006; Zordan et al., 2007; Hernday et al., 2013; Lohse et al., 2013; Hernday et al., 2016; Lohse and Johnson, 2016). Furthermore, ectopic *WOR1* expression can rescue opaque cell formation in all known mutant backgrounds that fail to spontaneously switch to the opaque cell type (Zordan et al., 2007; Du et al., 2012a; Lohse and Johnson, 2016). *WOR1* expression is repressed in white cells, where Wor1 protein levels have been found to be nearly undetectable (Huang et al., 2006; Srikantha et al., 2006; Zordan et al., 2006; Zordan et al., 2007; Lohse and Johnson, 2010). In opaque cells, *WOR1* is highly transcribed, and Wor1 protein levels have been found to accumulate to elevated levels (Huang et al., 2006; Srikantha et al., 2006; Zordan et al., 2006; Zordan et al., 2007; Lohse and Johnson, 2010). Stochastic white to opaque switching is thought to be the result of transcriptional noise within the white cell circuit that occasionally allows Wor1 levels to surpass a critical threshold necessary to induce the transition to the opaque state  (Srikantha et al., 2006; Hernday et al., 2010; Lohse and Johnson, 2010; Nobile et al., 2012; Guan and Liu, 2015; Horwitz et al., 2015; Lohse and Johnson, 2016; Lohse et al., 2016a; Tandonnet and Torres, 2017). Once established, the excited opaque cell circuit is stably maintained by a series of nested feedback loops, including a positive autoregulatory feedback loop generated by Wor1 binding to the upstream intergenic region of *WOR1* (Zordan et al., 2007; Hernday et al., 2013). This Wor1-induced positive feedback loop, along with other opaque specific binding interactions between the white and opaque regulators and their respective upstream intergenic regions, is proposed to be a central mechanism that mediates the epigenetic heritability of the opaque cell type (Huang et al., 2006; Srikantha et al., 2006; Zordan et al., 2006; Zordan et al., 2007; Lohse and Johnson, 2010; Wang et al., 2011; Hernday et al., 2013; Lohse et al., 2013; Lohse and Johnson, 2016; Hernday et al., 2016). Stochastic opaque to white switching is believed to occur when transcriptional noise causes Wor1 levels to drop below a critical threshold, thus leading to a collapse of the excited opaque cell transcriptional program and a return to the ground white cell transcriptional program (Srikantha et al., 2006; Zordan et al., 2006; Lohse and Johnson, 2010).

The core transcriptional circuit in white cells consists of a series of feed-forward loops that ultimately repress the expression of *WOR1* and *WOR2*, both of which are key players in the establishment and/or maintenance of the opaque cell type (Zordan et al., 2007). Efg1, Ahr1, and Ssn6 all contribute to the stability of the white cell circuit and are believed to directly or indirectly repress the expression of *WOR1* and *WOR2* (Zordan et al., 2007; Tuch et al., 2010; Hernday et al., 2013; Hernday et al., 2016). Deletion of *EFG1*, *AHR1*, or *SSN6* destabilizes the white cell circuit such that most, if not all, of the cells in the population transition to the opaque state (Sonneborn et al., 1999; Srikantha et al., 2000; Vinces et al., 2006; Vinces and Kumamoto, 2007; Zordan et al., 2007; Wang et al., 2011; Hernday et al., 2016). Czf1, Wor3, and Wor4 are capable of destabilizing the white cell circuit, and induced expression of *CZF1*, *WOR3*, or *WOR4* in white cells can promote white to opaque switching in a Wor1 dependent manner (Zordan et al., 2007; Hernday et al., 2013; Lohse et al., 2013; Lohse and Johnson, 2016). Interestingly, neither Czf1 nor Wor3 is required for the heritable maintenance of the opaque state once switching has occurred (Zordan et al., 2007; Lohse et al., 2013). Based on these results and the structure of the white cell regulatory circuit (**Figure 3A**), Czf1 and Wor4 are thought to destabilize the white cell type by directly and indirectly antagonizing the white cell stabilizing activities of Ssn6, Ahr1, and Efg1, and by inducing opaque promoting factors such as *WOR3*, thus introducing the transcriptional noise that leads to the stochastic activation of the *WOR1* positive feedback loop and the transition to the opaque state. In addition to repression of *WOR1* and *WOR2*, the white cell transcriptional program results in repression of opaque enriched transcripts (e.g. *WOR3* and *CZF1*) as well as the activation of white enriched transcripts (e.g. *EFG1*), thus creating a series of feed-forward loops that act to stabilize the white cell circuit and prevent activation of the opaque state (Zordan et al., 2007; Hernday et al., 2013; Lohse et al., 2013).

In contrast to the core transcriptional circuit of the white cell type (**Figure 3A**), the core transcriptional circuit of the opaque cell type is extensively intertwined (**Figure 3B**). All of the core switch regulators are active in opaque cells, and they are each found to bind to their own upstream intergenic regions, along with the upstream intergenic regions of most, if not all, of the other core switch regulators (**Figure 3B**) (Huang et al., 2006; Srikantha et al., 2006; Zordan et al., 2006; Zordan et al., 2007; Wang et al., 2011; Hernday et al., 2013; Lohse et al., 2013; Hernday et al., 2016; Lohse and Johnson, 2016). To highlight this point, 58 of the 64 possible binding interactions between the core switch regulators and their respective upstream intergenic regions are observed in opaque cells (**Data Sheet S1, Tab1**). Although the logic of the opaque transcriptional circuit has yet to be fully elucidated, the high degree of interconnectivity between the core opaque regulators likely contributes to the robustness, yet reversibility, of the opaque cell state. Similar to the white cell circuit, Wor1 is a critical player in the opaque cell circuit; however, it is the sustained high levels of *WOR1* expression, rather than its repression, that is required for the formation and stable maintenance of the opaque cell type (Huang et al., 2006; Srikantha et al., 2006; Zordan et al., 2006). Although not strictly required for the formation of an opaque cell, Wor2 and Wor4 also play important roles in the heritable maintenance of the opaque transcriptional program (Zordan et al., 2007; Lohse and Johnson, 2016). Strains lacking *WOR2* or *WOR4* are locked in the white cell type and fail to undergo spontaneous white to opaque switching, yet can be induced to form opaque cells by ectopic expression of *WOR1* (Frazer et al., 2020). These induced opaque cells, however, are unstable, and quickly revert to the white cell type when ectopic *WOR1* expression is repressed, indicating that Wor2 and Wor4 play essential roles in the heritability of opaque cells (Zordan et al., 2007). Interestingly, with the exception of Ahr1, all switch regulators discovered to date have been found to contain prion-like domains that enable liquid-liquid demixing and the formation of phase-separated condensates (Frazer et al., 2020). Several of the switch regulators, including Wor1 and Wor4, have been shown to undergo phase separation *in vitro*, and to form condensates at genomic loci *in vivo*, in a manner similar to the formation of mammalian super-enhancers (Frazer et al., 2020). Combined with the observation that many of the target genes bound by the switch regulators are flanked by unusually large upstream intergenic regions (Zordan et al., 2007; Hernday et al., 2013), and the discovery that specific residues within the Wor1 prion-like domain are required for condensate formation and white to opaque switching, it seems likely that these phase-separated condensates formed by the core switch regulators in opaque cells are critical factors that contribute to the formation and heritable maintenance of the opaque cell type.

## Regulation of the Commensal-Pathogen Transition

*C. albicans* typically exists as a commensal member of the healthy human microbiota. It can also transition into a pathogen in response to specific host environmental cues. In its pathogenic state, *C. albicans* can cause a wide range of infections, from acute to chronic superficial mucosal infections to severe and life-threatening disseminated bloodstream infections (Wenzel, 1995; Hube, 2004; Pappas et al., 2004). Although immunocompetent individuals with healthy and balanced microbiota are typically not adversely affected by *C. albicans*, immunocompromised individuals can suffer severe infections with significant morbidity and mortality (Wenzel, 1995; Nobile and Johnson, 2015). Understanding the genetic regulatory mechanisms that control the *C. albicans* commensal-pathogen transition has the potential to lead to the development of targeted therapeutic strategies against *C. albicans* in its pathogenic state, without affecting its commensal state and the delicate balance of the microbiota.

**Figure 4:**
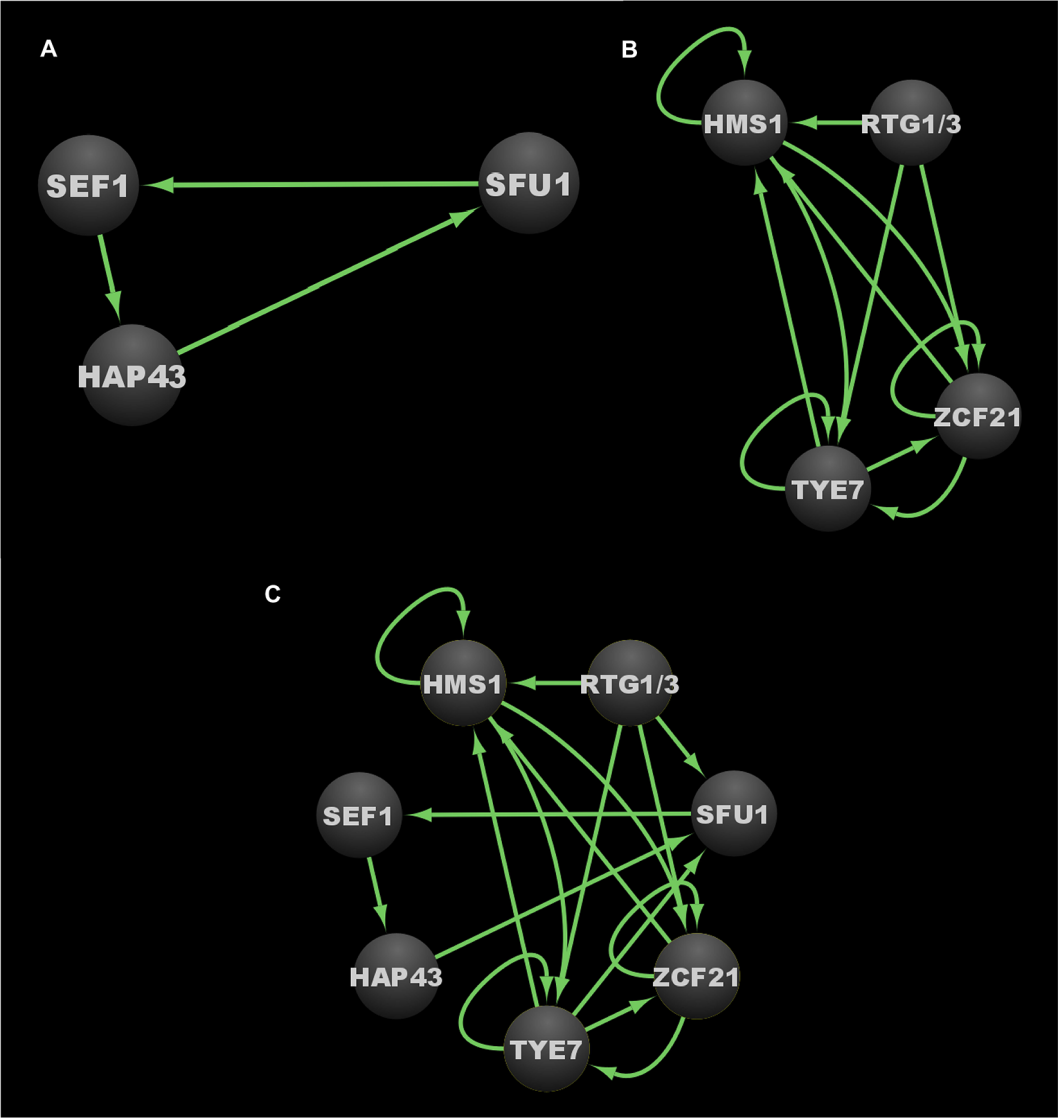
Transcriptional circuits controlling the *C. albicans* commensal-pathogen transition. **(A)** Transcriptional circuit controlling iron homeostasis. **(B)** Transcriptional circuit controlling proliferation in the host. **(C)** Overlayed transcriptional circuits controlling the commensal-pathogen transition. Ovals indicate each of the core regulators with their respective names. Arrows indicate direct binding events. Note that since Rtg1 and Rtg3 function as a heterodimer, and do not appear to bind DNA independently, they are represented as a single node in these regulatory circuit diagrams. See **Data Sheet S1** **, Tab4** for binding interactions. Data were derived from (Chen et al., 2011; Pérez et al., 2013). Figure was generated using Cytoscape (Shannon et al., 2003).

Two distinct *C. albicans* transcriptional networks controlling the commensal-pathogen transition were described in 2011 and 2013, one governing iron homeostasis, and the other governing proliferation in the host, respectively (see **Figure 4A** for the iron homeostasis circuit, **Figure 4B** for the proliferation in the host circuit, and **Figure 4C** for the combined commensal-pathogen overlayed circuits) (Chen et al., 2011; Pérez et al., 2013). As a commensal of the gastrointestinal (GI) tract, *C. albicans* is exposed to varying and often abundant levels of iron from food, and thus a tightly regulated transcriptional response is important for *C. albicans* to control iron assimilation and to avoid iron toxicity in the GI tract (McCance and Widdowson, 1938; Martin et al., 1987; Miret et al., 2003). On the other hand, when *C. albicans* causes a disseminated bloodstream infection, iron is extremely limiting, and to survive, *C. albicans* must conserve and scavenge iron from the bloodstream. Three transcriptional regulators, Sef1, Sfu1, and Hap43, were found to form a tightly knit transcriptional network, encompassing 214 downstream target genes (Chen et al., 2011). These three transcriptional regulators control iron homeostasis and were found to be essential for *C. albicans* to survive as both a commensal and as a pathogen within the mammalian host (Chen et al., 2011). Iron homeostasis in many other fungi (such as in other ascomycetes and the basidiomycete, *Cryptococcus neoformans*) is commonly regulated by a bipartite regulatory circuit composed of orthologs of Sfu1 and Hap43, where Sfu1 orthologs repress iron acquisition genes and *HAP43* orthologs, while Hap43 orthologs repress nonessential iron utilization genes and *SFU1* orthologs. This mutually repressive regulatory interaction between orthologs of Sfu1 and Hap43 in other fungi is significantly altered in *C. albicans* by the intercalation of Sef1 as a third player within this circuit (**Figure 4A**) (Chen et al., 2011). In *C. albicans*, Sfu1 directly represses *SEF1* and iron acquisition genes under iron replete conditions (Chen et al., 2011). In response to iron limitation, Sef1 serves to directly activate *HAP43* and iron uptake genes, while Hap43 directly represses *SFU1* and iron utilization genes (Chen et al., 2011). Although the roles for Hap43 in *C. albicans* are similar to those of other fungi, the reciprocal interaction between Sfu1 and *HAP43* is altered in *C. albicans* by the inclusion of Sef1, which serves as an intermediary between Sfu1 and *HAP43*. *C. albicans SEF1* and *SFU1* are differentially expressed between growth in the GI tract versus growth in the bloodstream (Chen et al., 2011), thus providing dual inputs into the circuit controlling iron acquisition and utilization. While both Sef1 and Sfu1 serve to promote commensalism in a mouse GI commensal model, only Sef1 is required for virulence in a mouse disseminated infection model (Chen et al., 2011). Interestingly, deletion of *SFU1* conferred a significant competitive advantage over wildtype cells in the disseminated infection model (Chen et al., 2011), indicating that Sfu1 serves not only to promote commensalism in the GI tract, but also to attenuate virulence in the bloodstream. (See **Table 3** for information on these three core transcriptional regulators in the commensal-pathogen transition.) Ultimately the *C. albicans* iron homeostasis circuit produces a well conserved transcriptional output consisting of increased iron uptake and reduced iron utilization in iron limited environments, and decreased iron uptake and increased iron utilization in iron replete conditions. Despite being well conserved in its transcriptional output, the iron homeostasis circuit appears to be uniquely evolved in *C. albicans* to control the delicate balance between its commensal and pathogenic growth states.

**Table 3:** Known transcriptional regulators with roles in the *C. albicans* commensal-pathogen transition.

Core Iron Homeostasis Transcriptional Regulators				
Orf19#	Name	Known commensal-pathogen-related process affected in mutant strain	Gene upstream intergenic region bound by one or more of the core regulators?	References
Orf19.681	Hap43	Iron Utilization	Yes	(Baek et al., 2008; Chen et al., 2011; Hsu et al., 2011)
Orf19.3753	Sef1	Iron Uptake	Yes	(Chen et al., 2011)
Orf19.4869	Sfu1	Iron Acquisition	Yes	(Lan et al., 2004; Chen et al., 2011)
**Core Host Proliferation Transcriptional Regulators**				
**Orf19#**	**Name**	**Known commensal-pathogen-related process affected in mutant strain**	**Gene upstream intergenic region bound by one or more of the core biofilm regulators?**	**References**
Orf19.921	Hms1	GI Colonization, Disseminated Infection	Yes	(Shapiro et al., 2012; Pérez et al., 2013)
Orf19.4722	Rtg1	GI Colonization, Disseminated Infection	Yes	(Jia et al., 1997; Pérez et al., 2013)
Orf19.2315	Rtg3	GI Colonization, Disseminated Infection	Yes	(Jia et al., 1997; Pérez et al., 2013)
Orf19.4941	Tye7	GI Colonization	Yes	(Pérez et al., 2013)
Orf19.4166	Zcf21	Disseminated Infection	Yes	(Pérez et al., 2013)

A subsequent study identified eight transcriptional regulators (Tye7, Orf19.3625, Lys144, Zcf21, Lys14, Hsm1, Rtg1, and Rtg3) that influence *C. albicans* proliferation in the commensal and/or pathogenic growth states (Pérez et al., 2013). These regulators were identified by screening a subset of the commonly used *C. albicans* TF mutant library (Homann et al., 2009) for defects in a commensal (GI colonization) mouse model and a pathogenic (disseminated infection) mouse model. This subset of the TF mutant library consisted of those mutant strains that revealed no phenotypes in a diverse panel of *in vitro* growth conditions, and was screened to identify transcriptional regulators that were specifically required for normal (wildtype) levels of growth in either of the two mouse models (Homann et al., 2009; Pérez et al., 2013). Of the eight regulators that were identified, six (Rtg1, Rtg3, Tye7, Hms1, Orf19.3625, and Lys144) were required for GI colonization, while five (Rtg1, Rtg3, Hms1, Lys14, and Zcf21) were required for robust growth in the disseminated infection model (Pérez et al., 2013). Overall, Tye7, Orf19.3625, and Lys144 were found to be specific to commensal colonization of the GI tract; Zcf21 and Lys14 were found to be specific to disseminated infections; and Rtg1, Rtg3, and Hms1 were found to be associated generally with growth in the host (Pérez et al., 2013). Based on genome-wide transcriptional profiling and chromatin immunoprecipitation data, seven of these regulators (Tye7, Lys144, Zcf21, Lys14, Hsm1, Rtg1, and Rtg3) were found to form a transcriptional network consisting of 808 directly bound target genes. Significant overlap was observed between the bound target genes of this network and those genes that were upregulated in the mouse GI model compared to growth *in vitro*. Orf19.3625 was excluded from this analysis as it is a predicted subunit of a histone remodeling complex, and thus was not considered to be a specific regulator within the commensal-pathogen network. In contrast to the transcriptional network defined by Sef1, Sfu1, and Hap43, which is primarily responsible for regulating genes involved in iron homeostasis (Chen et al., 2011), the transcriptional network defined by Tye7, Lys144, Zcf21, Lys14, Hsm1, Rtg1, and Rtg3 appears to primarily regulate genes involved in the acquisition and metabolism of carbon and nitrogen, as well as genes that encode transporters and cell surface proteins (Pérez et al., 2013). The binding profiles for Rtg1 and Rtg3 were observed to be identical, and thus they likely function as a heterodimer to bind DNA (Pérez et al., 2013), which is consistent with their orthologs in *Saccharomyces cerevisiae* (Liu and Butow, 2006). Of the 153 direct target genes in this network that are upregulated during GI colonization and disseminated infection, 108 of them are bound by the Rtg1/3 heterodimer (Pérez et al., 2013), highlighting the central role that Rtg1/3 plays in this network. We note that a subsequent study by the same group identified five transcriptional regulators that influence fitness in an oropharyngeal candidiasis model (Cup9, Zcf8, Zcf21, Zcf27, and Orf19.217), and identified a set of genes that are differentially regulated in response to deletion of *CUP9* (Meir et al., 2018). We did not include these data in our analyses since binding experiments that would be necessary to integrate these additional regulators into the commensal-pathogen transcriptional circuit have not been performed.

At the core of this commensal-pathogen transcriptional network lies a tightly interwoven regulatory circuit defined by the binding interactions between five of these transcriptional regulators (Hms1, Zcf21, Tye7, Rtg1, and Rtg3) and their respective upstream intergenic regions (**Figure 4B**). While Lys14 and Lys144 are clearly important for pathogenic and commensal growth, respectively, they are not integrated into the core transcriptional circuit and instead appear to function as auxiliary regulators. Interestingly, *RTG1* and *RTG3* are not regulated at the transcriptional level in response to growth in the GI tract and are not direct targets of any of the members of this commensal-pathogen transcriptional circuit (Pérez et al., 2013). Instead, Rtg1/3 seems to function as a major regulatory input into, rather than target of, this commensal-pathogen circuit. In *S. cerevisiae*, the Rtg1/3 heterodimer is known to be post-translationally modified and translocated into the nucleus in response to growth on poor nitrogen sources or mitochondrial dysfunction, suggesting that nitrogen assimilation and metabolic adaptation could be critical factors for the proliferation of *C. albicans* in the host (Liao and Butow, 1993; Jia et al., 1997; Liu and Butow, 2006). Hms1, which is also required for both commensal and pathogenic growth in the host, is known to be activated in response to elevated temperatures (Shapiro et al., 2012), indicating that temperature, along with nitrogen source(s), represent two critical environmental signals that influence the commensal and pathogenic growth programs of *C. albicans*. Zcf21 represses a variety of genes that encode key virulence factors, and plays a major role in pathogenesis by balancing the positive effects of these virulence factors during disseminated infection against the increased susceptibility to host immune system recognition and clearance that is correlated with their expression (Böhm et al., 2016). Finally, Tye7 has been implicated in the metabolism of carbohydrates, such as oligosaccharides and polysaccharides, as well as in the regulation of hyphal growth and biofilm formation (Askew et al., 2009; Bonhomme et al., 2011). (See **Table 3** for information on these five core transcriptional regulators in the commensal-pathogen transition.) Although both the iron homeostasis and the host proliferation transcriptional networks are critical to the ability of *C. albicans* to grow as a commensal and as a pathogen, there is limited interconnectivity between these networks at the level of the core regulators of each circuit (**Figure 4C**). *SFU1* serves as the sole point of integration between the two circuits, being bound by Rtg1/3 and Tye7. There are no binding interactions observed between the iron homeostasis regulators (Sef1, Sfu1, and Hap43) and the genes encoding the host proliferation regulators, suggesting that under certain growth conditions which alter the binding of Rtg1/3 and/or Tye7, the iron homeostasis circuit may function as a sub-circuit of the host proliferation circuit. Together, the transcriptional regulators involved in iron homeostasis and acquisition, and host proliferation, confer *C. albicans* with the ability to proliferate in different niches of the host as well as to transition between commensal and pathogenic states in response to changes in the host environment.

## Integration of Networks

**Figure 5:**
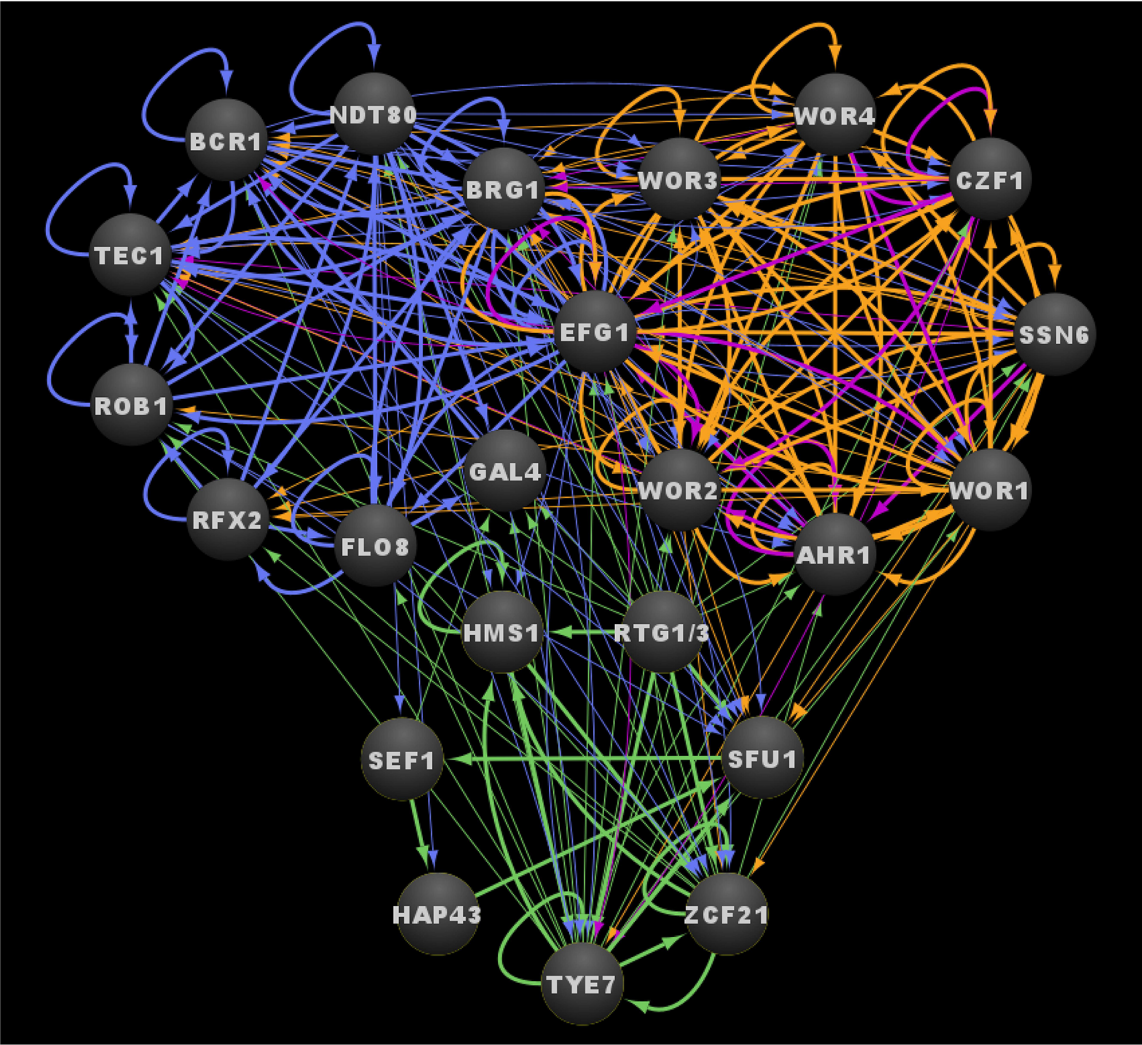
Integrated transcriptional circuits of *C. albicans* biofilm formation, the white-opaque switch and the commensal-pathogen transition. Ovals indicate each of the core regulators with their respective names. Arrows indicate direct binding events. See **Data Sheet S1** **, Tab4** for binding interactions. Data were derived from (Zordan et al., 2007; Chen et al., 2011; Nobile et al., 2012; Hernday et al., 2013; Lohse et al., 2013; Pérez et al., 2013; Fox et al., 2015; Hernday et al., 2016; Lohse and Johnson, 2016). Figure was generated using Cytoscape (Shannon et al., 2003).

In total, the three larger regulatory networks, consisting of the core regulators and all of their directly bound target genes involved in biofilm formation, the white-opaque phenotypic switch, and the commensal-pathogen transition in *C. albicans* encompass at least 1657 directly bound individual target genes, making up a little over 25% of genes in the entire genome (note that Flo8, Gal4, and Rfx2 were excluded from this analysis since there is not genome-wide chromatin immunoprecipitation data available for them) (**Data Sheet S1, Tab2**) (Zordan et al., 2007; Chen et al., 2011; Nobile et al., 2012; Hernday et al., 2013; Lohse et al., 2013; Pérez et al., 2013; Fox et al., 2015; Hernday et al., 2016; Lohse and Johnson, 2016). These three networks are highly intertwined, with 40% (667/1657) of the target genes shared between at least two of the networks, and 11% (188/1657) of the target genes shared between all three networks (**Data Sheet S1** **, Tab2**). This high degree of interconnectivity is even more pronounced at the level of the core transcriptional circuits that control these three networks, as is evident by the extensive binding interactions present between the core regulators themselves (**Figure 5** and **Data Sheet S1** **, Tab1**). Together, the twenty transcriptional regulators for which we have genome-wide chromatin immunoprecipitation data available form a total of 225 binding interactions within and between their core circuits, distributed roughly evenly between intra-circuit (49%) and inter-circuit (51%) interactions (note that the Rtg1/3 heterodimer is counted as a single regulator since neither subunit is known to bind independently) (**Data Sheet S1** **, Tab3**). The commensal-pathogen circuit and the biofilm circuit are highly intertwined with the regulators in the other circuits, with 66% and 59% inter-circuit interactions, respectively, while the opaque cell circuit appears to be much more isolated, with the majority (64%) of its interactions being intra-circuit (**Data Sheet S1** **, Tab3**). Perhaps the most striking example of integration between the circuits is exemplified by Ndt80 in the biofilm circuit, which binds to the upstream intergenic regions of 22 out of 24 of the core regulators (all but the upstream intergenic regions of *RTG1* and *RTG3*) (**Data Sheet S1** **, Tab1**). The percentage of inter-circuit binding events is highest for Tye7 (79%), Zcf21 (75%), Bcr1 (71%), Brg1 (67%), and Rtg1/3 (67%), accounting for at least two out of three binding events for each of these regulators within the three core circuits (**Data Sheet S1** **, Tab3**). At the opposite end of the spectrum, Hap43, Hms1, and Sfu1 are exclusive to the commensal-pathogen circuit. In addition, at least two thirds of the binding events observed for Wor3 (88%), Czf1 (75%), Rob1 (71%), Ahr1 (70%), and Wor4 (70%) within the three core circuits occur within their respective core circuits (**Data Sheet S1** **, Tab3**). Interestingly, the degree of Efg1 inter-circuit interaction is unique to the circuit within which it lies, where 61% inter-circuit interactions are observed for Efg1 in the biofilm circuit, while only 42% inter-circuit interactions are observed for Efg1 in the white-opaque circuit (**Data Sheet S1** **, Tab3**). *BRG1* is the most highly integrated target within the three circuits, where it is bound by seventeen of the twenty core regulators evaluated (leaving out Gal4, Rfx2, and Flo8, and considering Rtg1 and Rtg3 as a single regulator) (**Data Sheet S1** **, Tab1**). Overall, more than half (thirteen out of twenty-four) of the regulators that make up the three core circuits are bound by at least half (eleven or more) of the twenty core regulators evaluated (**Data Sheet S1** **, Tab1**). These rather striking numbers highlight the degree to which these circuits are intertwined, and these numbers are only likely to increase as additional core regulators are identified and incorporated into the three transcriptional circuits.

The extensive integration between these core transcriptional circuits appears to have significant functional relevance. For example, 14 of the 24 regulator genes discussed (*AHR1*, *BCR1*, *BRG1*, *CZF1*, *GAL4*, *HAP43*, *HMS1*, *RFX2*, *SEF1*, *SFU1*, *TEC1*, *WOR1*, *WOR3*, *ZCF21*) are differentially expressed by at least twofold between planktonic and biofilm growth conditions; of these fourteen genes, all but *GAL4* are upregulated in biofilms (**Data Sheet S1** **, Tab1**) (Nobile et al., 2012). A similar trend is observed during white-opaque switching, where eleven of the twenty-four regulator genes (*BRG1*, *CZF1*, *EFG1*, *GAL4*, *HMS1*, *RFX2*, *ROB1*, *TYE7*, *WOR1*, *WOR2*, *WOR3*) are differentially expressed by at least twofold between white and opaque cell types (**Data Sheet S1** **, Tab1**) (Tuch et al., 2010). The interactions between the biofilm circuit and the white-opaque circuit are particularly striking. All eight of the core white-opaque regulator genes are bound by at least four of the six core biofilm regulators, and six of the eight white-opaque regulator genes (all but *EFG1* and *WOR4*) are differentially expressed by twofold or more between planktonic and biofilm conditions (*WOR1*, *AHR1*, *CZF1*, and *WOR3* are upregulated by 3-, 5-, 8-, and 32-fold, respectively, while *WOR2* and *SSN6* are both downregulated by 2-fold) (**Data Sheet S1** **, Tab1**). Conversely, five of the nine core biofilm regulator genes are bound by at least four of the eight white-opaque regulators in opaque cells (*EFG1*, *BRG1*, *BCR1*, *TEC1*, and *RFX2* are bound by eight, eight, five, five, and four white-opaque regulators, respectively), and five of the nine biofilm regulator genes are differentially expressed by at least 2-fold between white and opaque cells (*BRG1* and *RFX2* are upregulated in opaque cells, while *EFG1*, *GAL4*, and *ROB1* are upregulated in white cells) (**Data Sheet S1** **, Tab1**). The commensal-pathogen circuit regulators are closely intertwined with the biofilm circuit; however, there is relatively little overlap between the overlayed white-opaque circuit and the overlayed commensal-pathogen circuit. Six of the eight commensal-pathogen regulator genes (all but *RTG1* and *RTG3*) are bound by at least one biofilm core regulator, half of which (*SFU1*, *TYE7*, and *ZCF21*) are bound by at least four of the biofilm regulators (**Data Sheet S1** **, Tab1**). All six of the commensal-pathogen regulator genes that are bound by biofilm regulators are differentially expressed by twofold or more between planktonic and biofilm conditions, with all but *TYE7* being upregulated in biofilms (**Data Sheet S1** **, Tab1**). In contrast to the high degree of functional interaction between the biofilm circuit and the overlayed commensal-pathogen circuit, only three of the eight commensal-pathogen regulator genes (*SFU1*, *TYE7*, and *ZCF21*) are bound by any of the white-opaque regulators, and of the three target genes, only *TYE7* is differentially expressed between white and opaque cells (upregulated twentyfold in opaque cells). The effect of growth under conditions relevant to the overlayed commensal-pathogen circuit (i.e. low iron or growth in the GI tract) is relatively limited when compared to the effects of biofilm formation and white-opaque switching. Upon growth in low iron, only the three regulator genes involved in iron homeostasis (*HAP43*, *SEF1*, *SFU1*) are differentially expressed (**Data Sheet S1** **, Tab1**) (Chen et al., 2011). While growth in the GI tract does affect the expression of core regulator genes in the other circuits, the impact of this expression is relatively limited, where *AHR1* and *TEC1* are upregulated and *ROB1* is downregulated in the GI tract versus growth *in vitro* (Rosenbach et al., 2010).

## Perspectives

The *C. albicans* transcriptional regulatory circuits controlling the developmental processes of biofilm formation, the white-opaque phenotypic switch, and the commensal-pathogen transition are individually tightly knit and we show that they are integrated together by extensive regulatory crosstalk between the core regulators that comprise each circuit. If we take into consideration all of the target genes in each of the larger transcriptional networks, each regulator controls individual subsets of target genes regulating distinct functions as well as subsets of target genes with functions in common with the other core regulators in each network. Strikingly, these three major transcriptional networks, together, encompass a little over 25% of genes in the entire genome, indicating that there is a high degree of functional redundancy across the networks. The complexity and functional redundancy of these network structures often make dissecting the logic of each network extremely challenging. The networks we discuss here in this review are overall structurally very similar to networks controlling complex transcriptional developmental processes in higher eukaryotes, such as the mammalian embryonic stem cell state (pluripotency) network (Boyer et al., 2005; Kim et al., 2008a). Given that mammals and *C. albicans* diverged from a common ancestor around 1.5 million years ago (Wang et al., 1999), it is notable that the structures of these independently evolved transcriptional networks are so similar. There are a couple hypotheses as to how these transcriptional networks could appear so structurally similar (Sorrells and Johnson, 2015). The first hypothesis is that these complex transcriptional networks represent the optimal solutions for organizing the biological processes they control (François and Hakim, 2004; Prill et al., 2005). The second hypothesis is that these transcriptional networks are not optimal solutions but are rather non-adaptive structures that have been retained over evolutionary time scales by purifying selection and are thus the result of high-probability evolutionary trajectories (Sorrells and Johnson, 2015). As we begin to discover and deconvolute complex transcriptional networks, we will begin to test these hypotheses and shed new light on the logic of these complex network structures.

## Author Contributions

Conceptualization: DR, MQ, AH, and CN. Formal Analysis: DR, MQ, AH, and CN. Investigation: DR, MQ, AH, and CN. Resources: AH, and CN. Data Curation: DR, MQ, AH, and CN. Writing—Original Draft Preparation: DR, MQ, AH, and CN. Writing—Review and Editing: DR, MQ, AH, and CN. Visualization: DR, AH, and CN. Supervision: AH and CN. Project Administration: AH and CN. Funding Acquisition: AH and CN. All authors contributed to the article and approved the submitted version.

## Funding

This work was supported by the National Institutes of Health (NIH) National Institute of Allergy and Infectious Diseases (NIAID) and National Institute of General Medical Sciences (NIGMS) awards R21AI125801 and R35GM124594, respectively, by a Pew Biomedical Scholar Award from the Pew Charitable Trusts, and by the Kamangar family in the form of an endowed chair to CN. This work was also supported by NIH NIAID award R15AI37975 to AH. DR was supported by the National Science Foundation (NSF) Graduate Research Fellowship Program (GRFP) award 1744620. The funders had no role in the study design, data collection and interpretation, or the decision to submit the work for publication.

## Conflict of Interest

CN is a cofounder of BioSynesis, Inc., a company developing inhibitors and diagnostics of biofilms.

The remaining authors declare that the research was conducted in the absence of any commercial or financial relationships that could be construed as a potential conflict of interest.

The reviewer AS declared a past co-authorship with one of the authors AH to the handling editor.

## Supplementary Material

Supplementary Data Sheet 1 Compilation and analysis of regulator binding interactions and target gene expression. **Tab1** labeled “Tab1_Combined Core Circuits” contains compiled genome-wide chromatin immunoprecipitation (ChIP-seq or ChIP-chip) and expression profiling (RNA-seq or microarray) data for the core circuit regulators and their respective target genes. ChIP data and RNA-seq data values are in log_2_ format. Biofilm regulators ChIP data and differential gene expression data were derived from (Nobile et al., 2012). White-opaque regulators ChIP data were derived from (Zordan et al., 2007; Hernday et al., 2013; Lohse et al., 2013; Hernday et al., 2016; Lohse and Johnson, 2016). White-opaque differential gene expression data were derived from (Tuch et al., 2010). Iron homeostasis regulators ChIP data and differential gene expression data were derived from (Chen et al., 2011). Host proliferation ChIP data were derived from (Pérez et al., 2013). Host proliferation differential gene expression data were derived from (Rosenbach et al., 2010). **Tab2** labeled “Tab2_Combined Networks” contains compiled genome-wide chromatin immunoprecipitation (ChIP-seq or ChIP-chip) and expression profiling (RNAseq or microarray) data for the core circuit regulators and all possible target genes in the *C. albicans* genome. ChIP data and RNA-seq data values are in log_2_ format. Biofilm regulators ChIP data and differential gene expression data were derived from (Nobile et al., 2012). White-opaque regulators ChIP data were derived from (Zordan et al., 2007; Hernday et al., 2013; Lohse et al., 2013; Hernday et al., 2016; Lohse and Johnson, 2016). White-opaque differential gene expression data were derived from (Tuch et al., 2010). Iron homeostasis regulators ChIP data and differential gene expression data were derived from (Chen et al., 2011). Host proliferation ChIP data were derived from (Pérez et al., 2013). Host proliferation differential gene expression data were derived from (Rosenbach et al., 2010). **Tab3** labeled “Tab3_Inter- vs Intra-circuit” contains an analysis of the genome-wide ChIP data from **Tab1**, tabulating the total number of bound targets for each of the regulators for which genome-wide binding data is available, and calculating the percentage of binding events that represent inter- versus intra-circuit binding interactions. **Tab4** labeled “Tab4_Cytoscape Interactions” contains a representation of the genome-wide ChIP data from **Tab1** in an interaction table format for visualization in Cytoscape (Shannon et al., 2003). This dataset was used to generate all of the circuit diagrams shown in **Figures 2** **–** **5**.
